# Betaine for the prevention and treatment of insulin resistance and fatty liver in a high-fat dietary model of insulin resistance in C57BL mice

**DOI:** 10.3389/fnut.2024.1409972

**Published:** 2024-07-25

**Authors:** Elango Kathirvel, Kengathevy Morgan, Olga V. Malysheva, Marie A. Caudill, Timothy R. Morgan

**Affiliations:** ^1^Research Healthcare Group, Veterans Administration Healthcare System, Long Beach, CA, United States; ^2^Division of Nutritional Sciences, Cornell University, Ithaca, NY, United States; ^3^Department of Medicine, University of California, Irvine, Irvine, CA, United States; ^4^Medical Healthcare Group, Veterans Administration Healthcare System, Long Beach, CA, United States

**Keywords:** betaine, insulin resistance, insulin receptor substrate, high fat diet, non-alcoholic fatty liver disease

## Abstract

**Aim:**

The aim was to investigate mechanisms by which betaine improves hepatic insulin signaling in a dietary mouse model of insulin resistance and fatty liver.

**Methods:**

C57BL 6J mice were fed a standard diet (SF), a standard diet with betaine (SFB), a nutritionally complete high fat (HF) diet, or a high fat diet with betaine (HFB) for 14 weeks. In a separate experiment, mice were fed high fat diet for 18 weeks, half of whom received betaine for the final 4 weeks. Activation of insulin signaling in the liver was assessed by western blot. Insulin signaling was also assessed in insulin resistant primary human hepatocytes treated with betaine.

**Results:**

As compared with SF, mice receiving HF diet were heavier, had more hepatic steatosis, and abnormal glucose tolerance test (GTT). Betaine content in liver and serum was 50% lower in HF than in SF; betaine supplementation restored serum and liver betaine content. Betaine treatment of HF reduced whole body insulin resistance as measured by GTT. Betaine treatment of HF increased tyrosine phosphorylation of insulin receptor substrate-1 and phosphorylation (activation) of Akt, and increased hepatic glycogen content. *In vitro*, betaine reversed insulin resistance in primary human hepatocytes by increasing insulin-stimulated tyrosine phosphorylation of IRS1 and of Akt.

**Conclusion:**

Betaine supplementation reduced whole body insulin resistance and increased activation of insulin signaling pathways in the liver in a mouse model of insulin resistance and fatty liver created by feeding a nutritionally complete high fat diet for 14 weeks. Betaine also reduced liver injury as assessed by ALT and by liver histology. *In vitro*, betaine reversed insulin resistance by increasing insulin-stimulated tyrosine phosphorylation of IRS1 and activation of downstream proteins in the insulin signaling cascade in insulin resistant primary human hepatocytes.

## Introduction

Insulin resistance, also known as decreased insulin sensitivity, is defined as reduced ability of insulin to activate insulin signaling pathways, resulting in increased fasting serum glucose and other abnormalities in glucose and fat metabolism. Insulin resistance is the underlying cause of type 2 diabetes and is associated with non-alcoholic fatty liver disease (1, 2). Insulin acts by binding to the transmembrane insulin receptor (IR), resulting in activation (autophosphorylation) of the intracellular subunit (insulin receptor-β [IRβ]), followed by phosphorylation of insulin receptor substrates, including insulin receptor substrate-1 (IRS1). Tyrosine phosphorylated IRS1 activates several downstream pathways that regulate cellular glucose and lipid metabolism, including Akt (also known as protein kinase B [PKB]) and glycogen synthase kinase three beta (GSK3β), the enzyme regulating hepatic glycogen synthesis.

The biochemical site of resistance to insulin, and the mechanisms contributing to resistance, in the liver are incompletely understood (3). Several *in vivo* and *in vitro* investigations report reduced insulin-mediated autophosphorylation of the β subunit of the insulin receptor (4). More commonly, reduced tyrosine phosphorylation of IRS1 has been reported in insulin resistance/diabetes.

Non-alcoholic fatty liver disease (NAFLD) is defined as excess accumulation of fat (triglycerides) in hepatocytes that is not due to ethanol consumption. NAFLD is the hepatic manifestation of the metabolic syndrome (1, 5) and is almost invariably associated with insulin resistance (2, 6, 7). Approximately one in four adults in the Western world has NAFLD and the prevalence is increasing in Asia (8, 9). An estimated 20% of patients with NAFLD have steatohepatitis, a type of liver injury characterized by accumulation of fat (steatosis), inflammation (“hepatitis”), and fibrosis, that can progress to cirrhosis and liver failure (8). NAFLD, and the associated inflammation and liver injury, is reversible with weight loss (10–12). Unfortunately, many patients are unable to lose sufficient weight to resolve NAFLD. Safe and effective pharmaceutical options to treat NAFLD are needed.

Betaine (trimethylglycine) is a cellular constituent with multiple roles in cellular metabolism (13). Betaine is the methyl donor for betaine-homocysteine S-methyltransferase (BHMT), the enzyme in the methionine cycle that converts homocysteine to methionine. Betaine is also an osmolyte, critical for maintenance of intracellular osmolality (14). More recently, betaine has been recognized as a cytoprotector and chemical chaperone (15). Betaine is available from dietary sources and is also synthesized in the liver from choline (16).

The role of betaine in insulin resistance and NAFLD is incompletely understood. Cross-sectional (8, 17, 18) and longitudinal studies (19) in humans have correlated low betaine blood levels with diabetes, insulin resistance, and NAFLD. Several clinical trials of betaine supplementation of humans with insulin resistance and/or fatty liver have reported improvements in fatty liver and insulin resistance (20, 21), although betaine supplementation failed to demonstrate benefit in other studies (22, 23). We (24) and others (25–27) have reported reduction (i.e., improvement) in insulin resistance and fatty liver with betaine treatment in animal models of high fat diet-induced obesity, insulin resistance/diabetes, and fatty liver. To date, a direct role for betaine on insulin signaling has not been explored.

The aim of the current study was to investigate mechanisms by which betaine might reduce (i.e., improve) hepatic insulin resistance *in vivo* in an animal model of diabetes and fatty liver. Mice were fed *ad lib* a nutritionally complete rodent diet containing 42% of calories from fat for 14 weeks. We measured the effect of betaine co-administration with the HF diet, as well as betaine administration after development of diabetes and fatty liver, on *in vivo* glucose metabolism and hepatic insulin signaling pathways. To evaluate a direct effect of betaine on insulin signaling, we measured several insulin signaling pathways *in vitro* in insulin resistant primary human hepatocytes with and without the addition of betaine.

## Materials and methods

### Animal experiments

Protocols were approved by the IACUC and other committees at the VA Long Beach Healthcare System responsible for overseeing the ethical treatment and use of animals prior to beginning the study and annually thereafter. Four-week-old male C57BL 6J were obtained from Jackson Laboratories, Bar Harbor, Maine. Six groups of mice were studied: control groups: mice were fed either standard diet, (SF, *n* = 10) or SF with betaine (SFB, *n* = 10) for 14 weeks; Prevention groups: mice were fed either a high fat diet, (pHF, *n* = 10) or HF with betaine (pHFB, *n* = 10) for 14 weeks; treatment groups: mice were fed HF diet for 14 weeks, after 14 weeks, half of the mice received betaine (tHFB, *n* = 10) and the other half received HF diet without betaine (tHF, *n* = 10) for an additional 4 weeks. Standard diet consisted of standard rodent chow (Teklad 8604), which was nutritionally complete and contained approximately 9% of calories from fat (by weight). The high fat diet (Teklad TD 88137) was nutritionally complete, containing approximately 42% of calories from fat, and was designed to mimic a “Western” diet. The nutrient composition of the diets is given in Table 1. Betaine hydrochloride (Sigma, St. Louis, MO, USA), 1% (wt/vol), was given *ad lib* in drinking water to the betaine groups. To measure hepatic insulin resistance, mice fasted overnight were injected 0.75 U/kg of insulin (Novolin, Novo Nordisk Inc., Princeton, NJ, USA) intraperitoneally 15 min before euthanizing by CO_2_ inhalation followed by cervical dislocation. Immediately following euthanasia, blood was drawn from the heart for serum biochemical tests. Liver and omental white adipose tissue (WAT) were removed, weighed, and snap frozen in liquid nitrogen, and stored at −80°C. Small slices of liver from two different lobes were fixed in 10% buffered formalin for histological examination.

**TABLE 1:** Macronutrient composition of the diets Teklad 8604 (SF) and TD 88137 (HF) (Diet was purchased from Teklad Diets, Madison, WI, USA).

Macronutrient	Teklad rodent diet	
	8604 (SF)	TD.88137 (HF)
Crude protein	24.3%	17.3%
Fat (ether extract)	4.7%	21.2%
Energy density	3.0 kcal/g	4.5 kcal/g
Calories from protein	32%	15.2%
Calories from fat	14%	42.0%
Calories from carbohydrate	54%	42.8%

### Insulin and glucose measurement

Two weeks before euthanasia mice were fasted overnight, and blood was drawn from the saphenous vein into heparinized capillary tubes. Glucose was measured using *ACCU-CHEK* Aviva glucometer (Roche, Mannheim, Germany). Plasma was separated by centrifugation and insulin level measured by ELISA using Ultra-sensitive rat insulin ELISA kit (Crystal Chem Inc., Downers Grove, IL, USA).

### Glucose tolerance test

Glucose tolerance test was performed as previously described (24). Briefly, one week before euthanasia, mice were fasted overnight, and fasting blood glucose was measured by tail snip. 1.5 mg glucose/g body weight was injected intraperitoneally, and tail blood glucose level was measured every 15 min for the first hour and every 30 min for the second hour using a glucometer (*ACCU-CHEK* Aviva, Roche).

### Primary human hepatocyte cell culture

Primary human hepatocytes in 24 well plates were purchased from Triangle Research Labs (HUM4049, Triangle Research Labs, Raleigh, NC, USA). These hepatocytes were isolated from a 35-year-old Caucasian male with a BMI of 28.5 and no history of alcohol and drug use. The hepatocytes were maintained in hepatocyte maintenance medium (Triangle Research Labs) for 12 h. Then, cells were incubated for 24 h in normal glucose (5.5 mM glucose) or in high glucose (30 mM glucose) with different concentrations of betaine (0.625, 1.25, 2.5, 5, 10, and 20 mM). This media did not contain FBS or insulin. After 24 h, cells were induced with 10 nM insulin for 10 min and then harvested in lysis buffer (Cell Signaling Technology). Protein was estimated by Dc protein assay (Bio-Rad), separated on an SDS-PAGE (Laemmli), and activation of various proteins was detected by immunoblotting.

### Histology

The formalin fixed liver samples were sectioned and stained with hematoxylin and eosin (H&E) and analyzed by a pathologist (S. W. French), in an unbiased fashion, for levels of inflammation, necrosis, and micro- and macrovesicular fat. Total pathology score was calculated as described previously (28).

### Serum alanine aminotransferase (ALT) measurements

ALT was measured as described previously (24). Blood was collected from the heart immediately after euthanasia. Serum was separated by centrifugation and diluted 1:1 with normal saline and analyzed in an automated analyzer (Hitachi Instruments, San Jose, California, USA).

### Hepatic triglycerides

Hepatic triglyceride was estimated using a triglyceride estimation kit (Abcam, Cambridge, MA, USA). Briefly, liver tissue (0.03–0.05 g) was homogenized in 5% TritonX100 and heated at 80–100°C for 4 min and cooled to room temperature. The homogenate was centrifuged at 6,000 rpm for 5 min and the triglyceride containing supernatant was analyzed as described in the manufacture’s protocol.

### Protein extraction, immunoprecipitation, SDS-PAGE, and western blotting

Protein extraction from liver: Protein was extracted from liver and from cell cultures as previously described (24). Liver tissue was homogenized in cell lysis buffer (Cell Signaling, Beverly, MA, USA) with protease and phosphatase inhibitors (Roche) and centrifuged at 10,000 rpm for 10 min at 4°C to remove debris. Protein extraction from cells: The cells were washed twice with ice cold PBS, stripped, and pelleted by centrifugation. The pellet was dissolved in lysis buffer (Cell Signaling) containing protease and phosphatase inhibitors (Roche). The cells in lysis buffer were sonicated and centrifuged at 10,000 rpm for 10 min at 4°C. Protein concentration in the supernatant was determined using the Dc protein assay (Bio-Rad Laboratories).

Immunoprecipitation: Immunoprecipitation was performed as previously described (29). Liver protein (∼500 μg) was incubated in 250 μL of cell lysis buffer (Cell Signaling) containing protease and phosphatase inhibitors (Roche) with antibodies (1:50 dilution) overnight in a rotary shaker at 4°C. Protein A agarose (20 μl) (Sigma) was added, and shaking was continued for 2 h. Agarose beads were washed with lysis buffer and Laemmli sample buffer (20 μL) was added to the agarose beads and denatured at 95°C for 4 min.

SDS-PAGE and Western Blotting: Denatured immunoprecipitated proteins were separated in 10% SDS-PAGE by electrophoresis (30) and transferred to nitrocellulose membrane. The membranes were blocked by 5% BSA in TBST (0.05 M Tris pH 7.6, 0.9% NaCl, 0.1% Tween-20). Primary antibodies (Cell Signaling Technology) for insulin receptor β (IRβ), tyrosine phosphorylated insulin receptor (p-tyr^1150/1154^-IRβ), insulin receptor substrate 1 (IRS1), tyrosine phosphorylated insulin receptor substrate 1 (p-tyr^895^-IRS1), serine phosphorylated insulin receptor substrate 1 (p-ser^307^-IRS1), protein kinase B (PKB, also known as Akt), serine phosphorylated protein kinase B (p-ser^473^-PKB/Akt), glycogen synthase kinase-3 β (GSK3 β), and serine phosphorylated glycogen synthase kinase-3 β (p-ser^9^-GSK3 β) were used at 1:1,000 dilutions in TBST containing 5% BSA overnight at 4°C. The membranes were incubated with a secondary antibody conjugated with horseradish peroxidase (Calbiochem, San Diego, CA, USA) at a dilution of 1:5,000 in 5% BSA in TBST for 1 h at room temperature and the specific proteins were detected with ECL Detection Kit (GE Healthcare, Buckinghamshire, UK). The relative density of the bands was measured using Un-Scan-it software Version 6.1 (Silk Scientific, Inc. Orem, UT, USA).

### Measurement of serum and hepatic betaine, dimethylglycine, and choline

Serum and hepatic betaine, dimethylglycine (DMG), and choline were measured as previously described (24). Extraction of betaine, dimethylglycine, and choline from mouse liver was carried out according to Mukherjee et al. (20) with minor modifications. Approximately 20 mg of frozen (−80°C) mouse liver was ground and mixed with acetonitrile (185 μL) containing 0.1% formic acid for protein precipitation. This was followed by the addition of a mixture of deuterium labeled internal standards (10 μL) containing 0.2 mM each of d9-choline, d9-betaine, and d6-dimethylglycine. After using a pellet mixer (VWR International, West Chester, PA) the samples were centrifuged at 10,600 × *g* for 15 min at 4°C. 150 μL of the supernatant was transferred to a separate vial from which 10 μL was injected into LC/MS/MS system (TSQ Quantum mass spectrometer, Thermo, San Jose, CA, USA) equipped with a refrigerated Accela autosampler (Thermo) and Accela pump with degasser (Thermo). Betaine, DMG, and choline were separated by HPLC using a Prevail silica column (150 × 2.1 mm, 5 um; Grace, Deerfield, IL) with a matching column guard (4.6 × 25 mm, 5 um). The mobile phase was run under isocratic conditions (500 μL/min) and consisted of acetonitrile (81%), ammonium formate (15 mM), and with 0.1% formic acid. The mass spectrometer was operated using a positive electron spray mode.

Quantification of betaine, dimethylglycine, and choline was performed by comparing samples with signals obtained from the standards using Xcalibur software (Thermo). Replicating the samples and in-house controls assured confidences in our results.

### Liver glycogen measurement

Liver glycogen was estimated as described previously (29). Briefly, liver (0.05 g) was homogenized in 5% TCA (1 mL) and centrifuged. Resulting supernatant (100 μL) was mixed with an equal volume of 10N KOH and boiled for 1 h. The solution was mixed with glacial acetic acid (50 μL) and H_2_O (750 μL), and 100 μL was added to a 200 μL of ice cold anthrone reagent. A blank was prepared by replacing the liver sample with 100 μL of 5% TCA. The absorbance was read at 650 nm. Glucose standard (0.125, 0.25, 0.5, 1, and 2 mg/mL) was used to calculate the amount of glucose (hydrolyzed glycogen) in liver samples.

### Statistical analysis

Data from the SF and prevention groups (SF, SFB, pHF, and pHFB) were analyzed by one-way ANOVA and compared within the group by Tukey’s test to determine significance level. Mice in the treatment groups (tHF and tHFB) were compared using Student’s *t*-test. The SIGMA STAT for Windows Version 10 statistical software was used (Systat Software, Inc., San Jose CA, USA). A *p*-value of < 0.05 was considered significant.

## Results

### Physical measurements

#### Body weight

**FIGURE 1:**
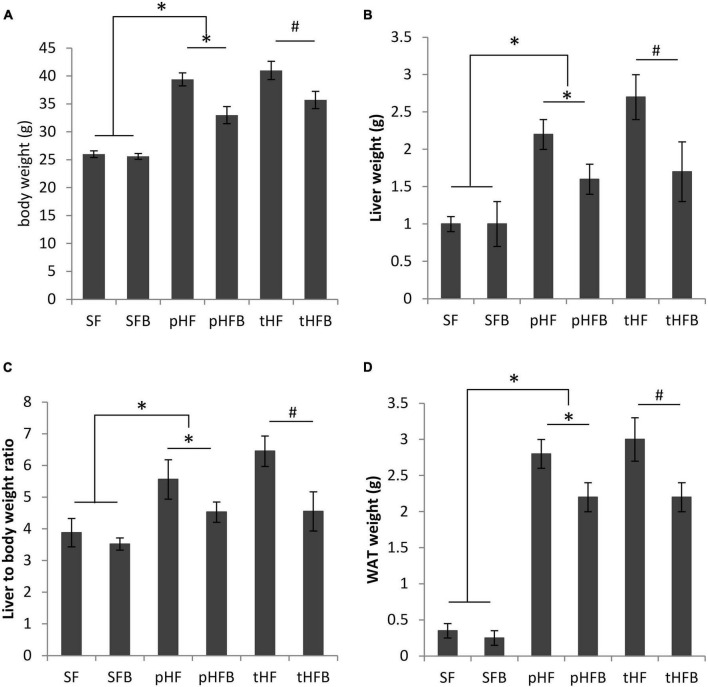
**(A)** Body weight, **(B)** liver weight, **(C)** liver to body weight ratio and **(D)** white adipose tissue (WAT) weight of distinct groups of mice. Values are mean ± SE of 10 animals per group. SF, SFB, pHF, and pHFB were analyzed by one-way ANOVA and compared by Tukey’s test (*p < 0.05). tHF and tHFB were analyzed by student’s *t*-test (#*p* < 0.05).

The body weight of SF and of SFB was not different (Figure 1A). Body weight in the pHF group was significantly higher than that in SF. Compared with the pHF group, body weight of pHFB group was 17% lower. Compared to tHF, the body weight of tHFB was 13% lower. Thus, betaine did not change body weight of standard fat fed mice but reduced body weight of mice receiving high fat diet.

#### Liver weight and liver weight /body weight ratio

The liver weight and liver to body weight ratios were similar in both SF and SFB (Figures 1B, C). The liver weight and liver to body weight ratio were higher in pHF as compared with pHFB or as compared with SF. The liver weight and the liver to body weight ratio was decreased in HF mice that received betaine (pHFB and tHFB) when compared with the respective HF mice that did not receive betaine (pHF and tHF).

#### Omental white adipose tissue weight

There was no difference in WAT weight between SFB and SF (Figure 1D). WAT weight was significantly increased in pHF as compared with pHFB or with SF. In the treatment group, WAT weight was significantly lower in tHFB as compared with tHF.

#### Hepatic betaine, dimethylglycine, and choline

**FIGURE 2:**
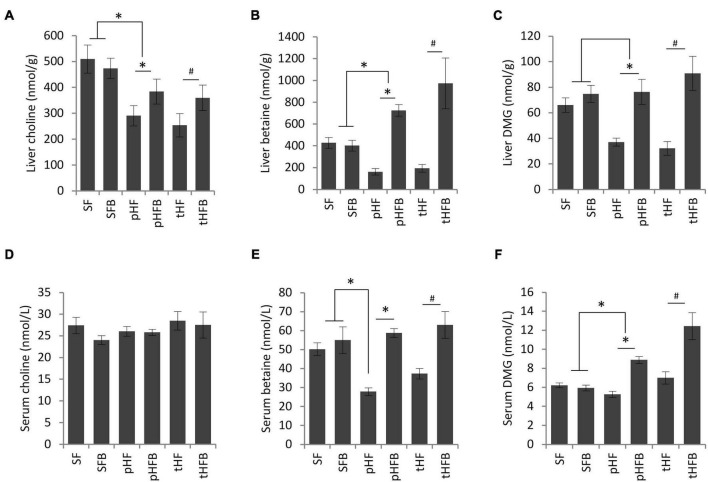
**(A)** Choline, **(B)** betaine, and **(C)** dimethylglycine levels in liver (values are in nmol/g); fasting serum choline **(D)**, betaine **(E)**, and dimethylglycine **(F)** levels in serum (values are nmol/L). Values are mean ± SE of 10 animals per group. SF, SFB, pHF, and pHFB were analyzed by one-way ANOVA and compared by Tukey’s test (*p < 0.05) and tHF and tHFB were compared using Student’s *t*-test (#*p* < 0.05). Fasting serum choline levels are not statistically different between groups.

Liver choline content was similar in SF and SFB. Liver choline content was higher in SF compared with the groups that were fed high fat diet (Figure 2A). Betaine supplementation increased choline levels in the livers of pHFB and tHFB compared to pHF and tHF. Betaine supplementation did not increase betaine content in the liver of mice fed standard diet (i.e., SFB vs. SF) (Figure 2B). Liver betaine content was approximately 50% lower in mice fed high fat diet (pHF) as compared with SF. When the high fat group was supplemented with betaine (pHFB), the level of betaine in the liver was three-fold higher when compared with pHF, to a level that was greater than the level found in SF. The liver betaine content of tHF was 50% lower than SF, and betaine content significantly increased in tHFB (Figure 2B). Hepatic DMG level was not statically different in SFB as compared with SF (Figure 2C). Similar to betaine, DMG level was significantly reduced in pHF compared with SF. Hepatic DMG levels increased significantly in tHFB and pHFB compared to tHF and pHF, respectively. Overall, hepatic choline, betaine and DMG levels decreased significantly with high fat diet; betaine supplementation returned these metabolites to normal or supranormal levels.

#### Serum betaine, dimethylglycine, and choline

Serum choline levels remained unchanged in all the groups (Figure 2D). Serum levels of betaine were similar in SF and SFB (Figure 2E). The serum betaine level was significantly lower in pHF and tHF than SF. Betaine supplementation increased serum betaine levels in pHFB and tHFB compared to the levels in tHF and pHF (Figure 2E). Serum level of DMG was not significantly different in SF, SFB, pHF and tHF. The level of serum DMG was significantly increased in pHFB and tHFB as compared with pHF and tHF, respectively (Figure 2F).

#### Histological and biochemical measurements of liver injury

##### Liver histology

**FIGURE 3:**
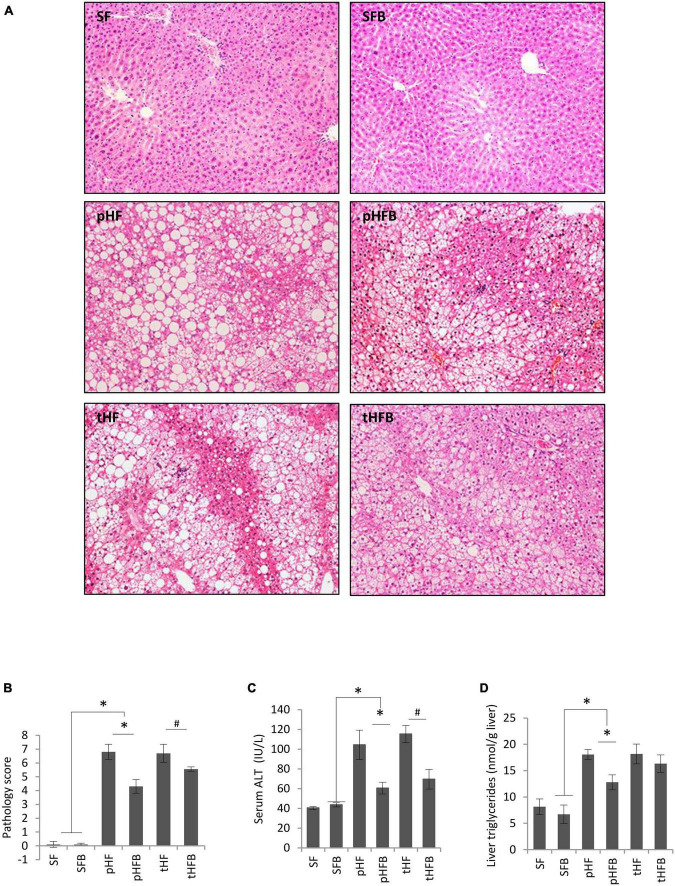
**(A)** liver histology (H&E, original ×200), **(B)** Total pathology score, **(C)** serum ALT, and **(D)** liver triglycerides. Values are mean ± SE of 10 animals per group. SF, SFB, pHF, and pHFB were analyzed by one-way ANOVA and compared by Tukey’s test (*p ≤ 0.05) and tHF and tHFB were compared using Student’s *t*-test (#*p* < 0.05).

The livers of SF and SFB were histologically normal (Figure 3A). The mice fed HF had microvesicular and macrovesicular fat, inflammation, and necrosis in the liver, similar to our prior report in Balb/c mice (29). The total pathology score, which is the sum of the scores for fat accumulation, inflammation, and necrosis, was significantly higher in pHF compared to SF (Figure 3B). The total pathology score decreased with betaine treatment, both in the prevention group (pHFB vs. pHF) and in the treatment group (tHFB vs. tHF).

##### Serum alanine aminotransaminase (ALT)

Serum ALT level was similar in SF and SFB (Figure 3C). ALT was higher in pHF than in SF. Betaine supplementation significantly decreased the ALT levels in pHFB and tHFB compared to pHF and tHF, respectively (Figure 3C).

##### Liver triglycerides

The hepatic triglyceride content in the SF and SFB did not differ (Figure 3D). Triglyceride content was significantly higher in pHF compared to SF. With betaine supplementation, liver triglyceride content was decreased in pHFB compared with pHF, but not in tHFB compared with tHF.

#### Measurements of *in vivo* insulin resistance

##### Fasting insulin and glucose

**FIGURE 4:**
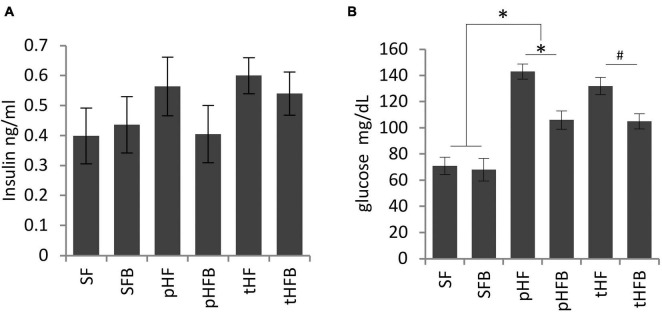
Fasting **(A)** serum insulin and **(B)** blood glucose levels. Values are mean ± SE of 10 animals per group. SF, SFB, pHF, and pHFB were analyzed by one-way ANOVA and compared by Tukey’s test (*p < 0.05) and tHF and tHFB were compared using Student’s *t*-test (#*p* < 0.05). Fasting insulin levels are not statistically different between the groups.

Fasting serum insulin concentration was similar in SF and SFB and was non-significantly higher in pHF than in SF (Figure 4A). Insulin concentration was decreased in pHFB, but the level was not statistically different than in pHF. Serum insulin level was similar in tHF and tHFB.

There was no difference in fasting glucose concentration in SF and SFB (Figure 4B). The fasting glucose concentration was increased in pHF compared to SF. The serum glucose levels were significantly reduced in the pHFB and in tHFB compared to pHF and tHF, respectively.

##### Glucose tolerance

**FIGURE 5:**
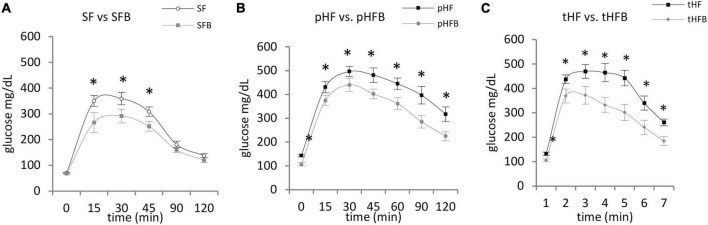
Glucose tolerance test. **(A)** SF vs. SFB, **(B)** pHF vs. pHFB, and **(C)** tHF vs. tHFB. Mice (*n* = 10 per group) were fasted overnight and then injected intraperitoneally with 1.5 mg glucose/g body weight. Blood glucose was measured every 15 min for first hour and at 30 min interval for the 2nd hour. The values are mean ± SE. Timepoints are compared using Student’s *t*-test and *p* < 0.05 (*) was considered significant.

SF and SFB had similar blood glucose levels at time zero. Glucose peaked at 30 min in both groups. Glucose level was significantly lower in SFB as compared with SF at 15, 30 and 45 min (Figure 5A). Glucose levels were similar at 2 h.

At time zero, glucose level in pHF was higher than in pHFB (Figure 5B). After intraperitoneal injection of glucose, the blood glucose levels peaked at 30 min in both groups. The glucose levels were significantly higher in pHF at all timepoints compared with pHFB. Results were similar in the treatment groups. Glucose was higher at time zero and at all subsequent time points in tHF compared with tHFB (Figure 5C).

#### Activation of hepatic insulin signaling proteins *in vivo* following insulin stimulation

##### Activation of insulin receptor

The activation of hepatic insulin signaling was estimated by measuring the level of phosphorylated insulin receptor by western blot 15 min after intraperitoneal insulin injection in mice that were fasted overnight. Betaine did not increase hepatic insulin receptor (IRβ) content in SFB vs. SF or in pHFB as compared with pHF (Figure 7). As shown by western blot analysis, betaine did not increase tyrosine phosphorylation of IRβ {i.e., no difference in phospho-tyrosine-IRβ between control and betaine-fed groups [i.e., SF vs. SFB or pHF vs. pHFB and tHF vs. tHFB (Figure 7)]}.

##### Activation of insulin receptor substrate-1

Following insulin binding to the insulin receptor, insulin receptor substrates are recruited and phosphorylated at tyrosine residues. Using specific antibodies, we measured the tyrosine phosphorylation of insulin receptor substrate-1 in the liver 15 min after intraperitoneal administration of insulin to fasted mice. IRS1 content was unchanged by HF or betaine administration (Figure 7). Tyrosine phosphorylation of IRS1 (activated) was increased in SFB compared with SF (Figure 7). In pHFB, the tyrosine phosphorylation of IRS1 was increased compared to pHF (Figure 7); similar results were observed when comparing tyrosine phosphorylation of IRS1 in tHF and tHFB.

##### Activation of Akt (PKB)

**FIGURE 6:**
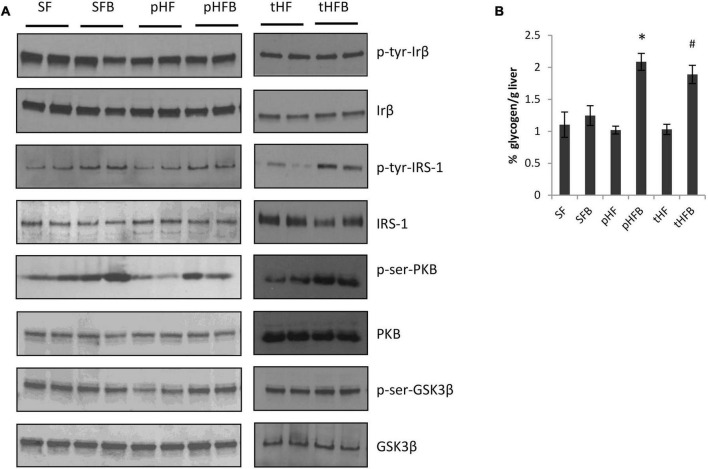
Immunoblot of liver proteins involved in the insulin signaling cascade **(A)**. **(B)** Hepatic glycogen content (mean ± SE; *n* = 10 per group). SF, SFB, pHF, and pHFB were analyzed by one-way ANOVA and compared by Tukey’s test (*p < 0.05) and tHF and tHFB were compared using Student’s *t*-test (#*p* < 0.05).

Following insulin-stimulated activation of IRS1, Pi3K is activated (phosphorylated) followed by activation (serine phosphorylation) of Akt. Similar to insulin-induced IRS1 activation, insulin-induced activation of Akt in fasted mice was increased in SFB as compared with SF (Figure 6A). The activation of Akt was decreased in pHF as compared with SF. The activation of Akt was increased in pHFB and tHFB as compared with pHF and tHF, respectively.

##### Activation of glycogen synthase kinase 3 beta (GSK3β) and liver glycogen content

Glycogen synthase kinase 3 beta regulates hepatic glycogen synthesis (that is, activated [serine phosphorylated] GSK3β stimulates glycogen synthesis). Insulin induced activation of GSK3β was similar in SF and SFB (Figure 6A). The activation of GSK3β was decreased in pHF when compared with SF. The addition of betaine in pHF (i.e., pHFB) resulted in greater activation of GSK3β when compared with pHF. Insulin-induced activation of GSK3β in the liver was similar in tHFB as compared with tHF. Glycogen content in HF groups fed betaine (pHFB and tHFB) was significantly increased as compared with their control groups (pHF and tHF) (Figure 6B). Glycogen content in SFB was not increased when compared with SF.

#### Activation of insulin signal proteins in insulin resistant primary hepatocytes

**FIGURE 7:**
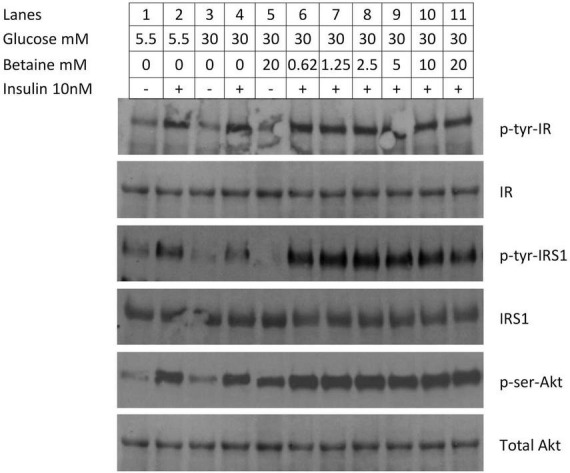
Immunoblot of proteins involved in insulin signaling in primary human hepatocytes. Insulin resistance was created by incubating hepatocytes with 30 mM glucose for 24 h. In the first four lanes demonstrate reduced activation of enzymes in the insulin signaling cascade. Cells were cultured in normal (5.5 mM) glucose (lanes 1&2) or high (30 mM) glucose (3&4) and induced with 10 mM insulin for 10 min (lanes 2&4). In the second set of experiments (lanes 5–11), the effect of betaine on insulin resistant human hepatocytes was evaluated. Insulin resistant cells were incubated with betaine alone (without insulin, lane 5) or with various concentrations of betaine (0.63 mM through 20 mM; lanes 6–11) for 24 h, followed by induction with 10 mM insulin for 10 min.

Primary human hepatocytes were made insulin resistant by growing in 30 mM glucose for 24 h and the effect of betaine on insulin signaling was evaluated. High glucose concentration induced insulin resistance, as shown by decreased insulin induced tyrosine phosphorylation of insulin receptor substrate-1 (Figure 7, lane 4) when compared to normal glucose (Figure 7, lane 2). The phosphorylation of insulin receptor-β following the addition of insulin was not different between normal and insulin resistant conditions (lane 4 vs. lane 2) and was not altered by incubation with betaine for 24 h (lane 4 vs. lanes 6–11). Insulin induced tyrosine phosphorylation of insulin receptor substrate-1 and serine phosphorylation of Akt were increased with betaine treatment (Figure 7, lanes 6–11) as compared with insulin resistant cells not treated with betaine (lane 4). Betaine alone, without insulin, did not activate IRS1 but did modestly activate Akt in insulin resistant cells (lane 5 vs. lane 3). We did not test the effect of betaine on insulin signaling in primary human hepatocytes that were maintained in 5.5 mM glucose (i.e., not insulin resistant).

## Discussion

This study evaluated the effect of oral betaine on whole body insulin resistance and on hepatic insulin signaling in a high fat dietary model of insulin resistance in C57BL 6J mice, the standard strain of mice used for diabetes research. Mice were fed a nutritionally complete diet containing either 9% of calories from fat (standard rodent chow) or 42% of calories from fat, a rodent diet designed to mimic a “Western” diet. Although fasting insulin was not significantly elevated, mice receiving HF diet had higher fasting glucose and were heavier than mice receiving SF, similar to prior reports of high fat diet-induced diabetes in C57BL/6 mice (25). Mice fed HF diet also had reduced insulin-induced hepatic tyrosine phosphorylation of IRS1, reduced phosphorylation (activation) of Akt, and reduced phosphorylation (activation) of GSK3β, the enzyme regulating glycogen storage. Thus, this HF dietary model had several biochemical findings typical of hepatic insulin resistance in type 2 diabetes (3).

Our findings confirm previously reported effects of betaine on liver injury and glucose metabolism and report new information on several insulin-activated metabolic pathways *in vivo* and *in vitro*. We and others (24, 25) have reported that betaine treatment of mice fed a high fat diet improves glucose tolerance test, reduces liver injury, and reduces hepatic steatosis, improvements that were largely confirmed in the current investigation. We did not measure insulin sensitivity, which Ejaz et al. (25) reported to be improved with betaine feeding to mice fed a high fat diet (45% calories from fat) for 16 weeks as assessed by insulin tolerance testing. However, the numerically lower fasting serum insulin level along with the significantly lower fasting glucose level, the two components of the human QUICKI measurement of insulin sensitivity, suggest that mice receiving HFB had greater whole body insulin sensitivity than mice receiving HF.

Similar to prior reports in humans with prediabetes (19, 23) and in animal models of dietary-induced diabetes (24, 25), the serum level of betaine was reduced in HF-fed mice when compared with mice fed SF. Betaine supplementation of mice fed HF restored serum betaine levels to the level in SF, but not to supranormal levels, as reported by Ejaz et al. (25). Although we did not measure dietary betaine consumption, our high fat diet contained a greater amount of betaine (per gram diet), making reduced betaine consumption unlikely to explain the reduced serum betaine level in HF when compared with SF. In a model similar to ours, in which C57BL/6 mice were fed ad lib a diet containing 45% of calories from fat for 16 weeks, the calculated dietary intake of betaine was comparable among mice fed the HF diet and mice fed SF (25).

Mice fed a HF diet had a reduction in the hepatic content of betaine and dimethylglycine as compared with SF, findings that we and others have noted (24, 25). The mechanism of the decreased hepatic levels of betaine and its metabolite is not known. Two possibilities include increased conversion of homocysteine to methionine and increased synthesis of very low density lipoproteins (VLDL) for lipid export. Both pathways consume betaine and would theoretically decrease serum and liver betaine levels in HF mice. Betaine supplementation led to supranormal levels of betaine in the liver of pHFB and tHFB when compared with SFB. We (24) and others (25) have reported an increased level of betaine in the liver of betaine-supplemented HF mice as compared with SF or HF in slightly different models of diet-induced obesity/fatty liver/insulin resistance. The increased concentration of betaine in the liver of pHFB in conjunction with a reduction in insulin resistance, as shown by improvements fasting glucose and the glucose tolerance test, suggest that higher hepatic levels of betaine may be important in maintaining hepatic insulin sensitivity or reducing hepatic fat accumulation.

Betaine administration for 4 weeks to mice with established fatty liver and insulin resistance created by feeding a high fat diet for 14 weeks (i.e., “treatment” studies) led to improvement in glucose tolerance test, ALT, and hepatic insulin signaling pathways. These findings support our results of co-administration of betaine and a HF diet (“prevention” studies reported above) and suggest the possibility of betaine as a treatment for non-alcoholic fatty liver disease and, potentially, for insulin resistance.

We evaluated hepatic insulin signaling in HF mice by measuring the level of activated proteins in the insulin signaling pathway shortly after administration of insulin to fasted mice. Betaine did not alter the amount of insulin receptor nor the amount of insulin-induced tyrosine phosphorylation of the insulin receptor in HF mice. However, betaine improved (nearly normalized) insulin-induced activation of IRS1 and Akt in mice fed HF diet in both the prevention (pHFB) and in the treatment (tHFB) groups. Betaine treatment of pHF mice (i.e., pHFB) also increased activation of GSK3β and increased hepatic glycogen content. In summary, betaine treatment reversed several biochemical abnormalities in the hepatic insulin signaling cascade related to glucose disposition in the liver of HF mice, beginning with increased activation of IRS1, and including increased activation of Akt and GSK3β, and increased glycogen content.

This is the first study of the effect of betaine administration on glucose metabolism, hepatic pathology, and hepatic insulin signaling pathways in mice fed a standard fat diet (containing ∼9% of calories from fat). Betaine administration did not alter the serum or the hepatic level of betaine, suggesting that hepatic betaine uptake may be regulated, and that betaine supplementation does not invariably elevate serum or hepatic betaine level. Also, betaine administration to mice receiving SF did not alter fasting glucose or fasting insulin although betaine supplementation did lead to a lower peak and more rapid reduction in serum glucose in the glucose tolerance test. Betaine administration to SF (i.e., SFB) resulted in greater activation of IRS1 and Akt in the liver following insulin stimulation, although there was no increase in GSK3β activation nor in hepatic glycogen content. In summary, betaine feeding to mice fed SF diet did not increase betaine levels in the serum or liver and did not alter fasting glucose or insulin levels. However, betaine led to more rapid improvement in serum glucose in the GTT and greater activation of insulin signaling proteins in the liver suggesting that betaine may increase insulin sensitivity in mice fed SF diet.

We examined the effect of betaine on the insulin signaling cascade in insulin resistant primary human hepatocytes. Incubation in high glucose medium for 24 h produced insulin resistance, as measured by decreased insulin-induced tyrosine phosphorylation (activation) of IRS1. The addition of various concentrations of betaine to the culture medium for 24 h, in the presence of continued high concentrations of glucose (30 mM), increased insulin sensitivity as measured by increased tyrosine phosphorylation of IRS1 and serine phosphorylation of Akt. Thus, betaine can reverse insulin resistance *in vitro* in insulin resistant primary human hepatocytes. The site of betaine’s action in normalizing insulin resistance appears to be at the level of tyrosine phosphorylation of IRS1, but not at the phosphorylation of insulin receptor nor at the amount of insulin receptor. The mechanism by which betaine increases IRS1 tyrosine phosphorylation is not known.

In summary, feeding a high fat diet to C57BL 6J mice resulted in overweight mice with biochemical findings of insulin resistance, including increased fasting glucose, reduced clearance of glucose in a glucose tolerance test, histological fatty liver, and hepatic biochemical evidence of reduced activation of the insulin signaling cascade after insulin stimulation. Betaine administration with the high fat diet to prevent insulin resistance, as well as for 4 weeks to treat established fatty liver and insulin resistance, improved histological liver injury, improved glucose tolerance test, and improved hepatic insulin sensitivity as measured by insulin-stimulated activation of IRS1, Akt, and GSK3β. High fat diet reduced betaine in the serum and liver while betaine supplementation increased serum and liver betaine to normal or supranormal levels. Betaine supplementation to mice fed SF had essentially no effect on hepatic histology, hepatic betaine level, or insulin resistance, other than increasing clearance of glucose in the glucose tolerance test and greater hepatic activation of IRS1 and Akt following insulin stimulation. Betaine improved activation of IRS1 and Akt *in vitro* in insulin resistant primary human hepatocytes, suggesting a direct effect of betaine on insulin sensitivity. Although the mechanism by which betaine increases activation of the insulin signaling cascade in insulin resistant primary human hepatocytes remains to be determined, betaine appears to act at the level of IRS1 activation (i.e., tyrosine phosphorylation), not at the level of insulin receptor quantity or activation (i.e., phosphorylation).

Human studies of betaine as a treatment for insulin resistance and/or NAFLD are conflicting. Given the current study, and the relatively small size of the existing human clinical trials, additional trials of betaine in humans with insulin resistance and/or NAFLD should be considered.

## Data availability statement

The original contributions presented in this study are included in this article/supplementary material, further inquiries can be directed to the corresponding author.

## Ethics statement

The animal study was approved by the Institutional Animal Care and Use Committee, VA Long Beach Healthcare System 5901 E. Seventh Street Long Beach, CA 90822, USA. The study was conducted in accordance with the local legislation and institutional requirements.

## Author contributions

EK: Conceptualization, Data curation, Formal analysis, Investigation, Methodology, Resources, Software, Writing – original draft, Writing – review & editing. KM: Conceptualization, Investigation, Methodology, Project administration, Resources, Supervision, Writing – original draft, Writing – review & editing. OM: Data curation, Investigation, Methodology, Writing – review & editing. MC: Data curation, Investigation, Methodology, Writing – review & editing. TM: Conceptualization, Formal analysis, Funding acquisition, Investigation, Methodology, Project administration, Resources, Supervision, Writing – original draft, Writing – review & editing.

## References

[1] Byrne C Targher G. NAFLD: A multisystem disease. *J Hepatol.* (2015) 62:S47–64. 25920090 10.1016/j.jhep.2014.12.012

[2] Lonardo A Lugari S Ballestri S Nascimbeni F Baldelli E Maurantonio M. A round trip from nonalcoholic fatty liver disease to diabetes: Molecular targets to the rescue? *Acta Diabetol.* (2019) 56:385–96. 10.1007/s00592-018-1266-0 30519965

[3] Santoleri D Titchenell P. Resolving the paradox of hepatic insulin resistance. *Cell Mol Gastroenterol Hepatol.* (2019) 7:447–56. 30739869 10.1016/j.jcmgh.2018.10.016 PMC6369222

[4] Youngren J. Regulation of insulin receptor function. *Cell Mol Life Sci.* (2007) 64:873–91. 17347799 10.1007/s00018-007-6359-9 PMC11135994

[5] Sookoian S Pirola C. Review article: Shared disease mechanisms between non-alcoholic fatty liver disease and metabolic syndrome – translating knowledge from systems biology to the bedside. *Aliment Pharmacol Ther.* (2019) 49:516–27. 10.1111/apt.15163 30714632

[6] Sakurai Y Kubota N Yamauchi T Kadowaki T. Role of insulin resistance in MAFLD. *Int J Mol Sci.* (2021) 22:4156. 10.3390/ijms22084156 PMC8072900 33923817

[7] Smith B Adams L. Nonalcoholic fatty liver disease and diabetes mellitus: Pathogenesis and treatment. *Nat Rev Endocrinol.* (2011) 7:456–65. 21556019 10.1038/nrendo.2011.72

[8] Estes C Razavi H Loomba R Younossi Z Sanyal A. Modeling the epidemic of nonalcoholic fatty liver disease demonstrates an exponential increase in burden of disease. *Hepatology.* (2018) 67:123–33. 10.1002/hep.29466 28802062 PMC5767767

[9] Younossi Z. Non-alcoholic fatty liver disease – a global public health perspective. *J Hepatol.* (2019) 70:531–44. 30414863 10.1016/j.jhep.2018.10.033

[10] Romero-Gomez M Zelber-Sagi S Trenell M. Treatment of NAFLD with diet, physical activity and exercise. *J Hepatol.* (2017) 67:829–46. 28545937 10.1016/j.jhep.2017.05.016

[11] Vilar-Gomez E Martinez-Perez Y Calzadilla-Bertot L Torres-Gonzalez A Gra-Oramas B Gonzalez-Fabian L Weight loss through lifestyle modification significantly reduces features of nonalcoholic steatohepatitis. *Gastroenterology.* (2015) 149:367–78.e5; quiz e14–5. 25865049 10.1053/j.gastro.2015.04.005

[12] Kenneally S Sier J Moore J. Efficacy of dietary and physical activity intervention in non-alcoholic fatty liver disease: A systematic review. *BMJ Open Gastroenterol.* (2017) 4:e000139. 10.1136/bmjgast-2017-000139 PMC5508801 28761689

[13] Day C Kempson S. Betaine chemistry, roles, and potential use in liver disease. *Biochim Biophys Acta.* (2016) 1860:1098–106. 26850693 10.1016/j.bbagen.2016.02.001

[14] Hoffmann L Brauers G Gehrmann T Haussinger D Mayatepek E Schliess F Osmotic regulation of hepatic betaine metabolism. *Am J Physiol Gastrointest Liver Physiol.* (2013) 304:G835–46. 23449672 10.1152/ajpgi.00332.2012

[15] Figueroa-Soto C Valenzuela-Soto E. Glycine betaine rather than acting only as an osmolyte also plays a role as regulator in cellular metabolism. *Biochimie.* (2018) 147:89–97. 10.1016/j.biochi.2018.01.002 29366935

[16] Zeisel S. Metabolic crosstalk between choline/1-carbon metabolism and energy homeostasis. *Clin Chem Lab Med.* (2013) 51:467–75. 10.1515/cclm-2012-0518 23072856 PMC3624053

[17] Konstantinova S Tell G Vollset S Nygard O Bleie O Ueland P. Divergent associations of plasma choline and betaine with components of metabolic syndrome in middle age and elderly men and women. *J Nutr.* (2008) 138:914–20. 10.1093/jn/138.5.914 18424601

[18] Sookoian S Puri P Castano G Scian R Mirshahi F Sanyal A Nonalcoholic steatohepatitis is associated with a state of betaine-insufficiency. *Liver Int.* (2017) 37:611–9. 10.1111/liv.13249 27614103

[19] Walford G Ma Y Clish C Florez J Wang T Gerszten R Metabolite profiles of diabetes incidence and intervention response in the diabetes prevention program. *Diabetes.* (2016) 65:1424–33. 10.2337/db15-1063 26861782 PMC4839205

[20] Mukherjee S Bernard T Kharbanda K Barak A Sorrell M Tuma D. Impact of betaine on hepatic fibrosis and homocysteine innonalcoholic steatohepatitis: A prospective, cohort study. *Open Transl Med J.* (2011) 3:1–4.

[21] Miglio F Rovati L Santoro A Setnikar I. Efficacy and safety of oral betaine glucuronate in non-alcoholic steatohepatitis. A double-blind, randomized, parallel-group, placebo-controlled prospective clinical study. *Arzneimittelforschung.* (2000) 50:722–7. 10.1055/s-0031-1300279 10994156

[22] Abdelmalek M Sanderson S Angulo P Soldevila-Pico C Liu C Peter J Betaine for nonalcoholic fatty liver disease: Results of a randomized placebo-controlled trial. *Hepatology.* (2009) 50:1818–26. 19824078 10.1002/hep.23239

[23] Grizales A Patti M Lin A Beckman J Sahni V Cloutier E Metabolic effects of betaine: A randomized clinical trial of betaine supplementation in prediabetes. *J Clin Endocrinol Metab.* (2018) 103:3038–49. 10.1210/jc.2018-00507 29860335 PMC6692715

[24] Kathirvel E Morgan K Nandgiri G Sandoval B Caudill M Bottiglieri T Betaine improves nonalcoholic fatty liver and associated hepatic insulin resistance: A potential mechanism for hepatoprotection by betaine. *Am J Physiol Gastrointest Liver Physiol.* (2010) 299:G1068–77. 10.1152/ajpgi.00249.2010 20724529 PMC2993168

[25] Ejaz A Martinez-Guino L Goldfine A Ribas-Aulinas F De Nigris V Ribo S Dietary betaine supplementation increases Fgf21 levels to improve glucose homeostasis and reduce hepatic lipid accumulation in mice. *Diabetes.* (2016) 65:902–12. 10.2337/db15-1094 26858359 PMC4806659

[26] Song Z Deaciuc I Zhou Z Song M Chen T Hill D Involvement of AMP-activated protein kinase in beneficial effects of betaine on high-sucrose diet-induced hepatic steatosis. *Am J Physiol Gastrointest Liver Physiol.* (2007) 293:G894–902. 10.1152/ajpgi.00133.2007 17702954 PMC4215798

[27] Wang Z Yao T Pini M Zhou Z Fantuzzi G Song Z. Betaine improved adipose tissue function in mice fed a high-fat diet: A mechanism for hepatoprotective effect of betaine in nonalcoholic fatty liver disease. *Am J Physiol Gastrointest Liver Physiol.* (2010) 298:G634–42. 10.1152/ajpgi.00249.2009 20203061 PMC2867421

[28] Morgan K Uyuni A Nandgiri G Mao L Castaneda L Kathirvel E Altered expression of transcription factors and genes regulating lipogenesis in liver and adipose tissue of mice with high fat diet-induced obesity and nonalcoholic fatty liver disease. *Eur J Gastroenterol Hepatol.* (2008) 20:843–54. 10.1097/MEG.0b013e3282f9b203 18794597

[29] Kathirvel E Morgan K French S Morgan T. Overexpression of liver-specific cytochrome P4502E1 impairs hepatic insulin signaling in a transgenic mouse model of nonalcoholic fatty liver disease. *Eur J Gastroenterol Hepatol.* (2009) 21:973–83. 10.1097/MEG.0b013e328328f461 19307976

[30] Laemmli U. Cleavage of structural proteins during the assembly of the head of bacteriophage T4. *Nature.* (1970) 227:680–5. 5432063 10.1038/227680a0

